# Histone Deacetylase Inhibitors (HDACi) Cause the Selective Depletion of Bromodomain Containing Proteins (BCPs)* [Fn FN2]

**DOI:** 10.1074/mcp.M114.042499

**Published:** 2015-03-09

**Authors:** Marie-Therese Mackmull, Murat Iskar, Luca Parca, Stephan Singer, Peer Bork, Alessandro Ori, Martin Beck

**Affiliations:** From the ‡European Molecular Biology Laboratory, Structural and Computational Biology Unit, Meyerhofstrasse 1, 69117 Heidelberg, Germany;; §Institute of Pathology, University Hospital Heidelberg, Im Neuenheimer Feld 224, 69120 Heidelberg, Germany

## Abstract

Histone deacetylases (HDACs) and acetyltransferases control the epigenetic regulation of gene expression through modification of histone marks. Histone deacetylase inhibitors (HDACi) are small molecules that interfere with histone tail modification, thus altering chromatin structure and epigenetically controlled pathways. They promote apoptosis in proliferating cells and are promising anticancer drugs. While some HDACi have already been approved for therapy and others are in different phases of clinical trials, the exact mechanism of action of this drug class remains elusive. Previous studies have shown that HDACis cause massive changes in chromatin structure but only moderate changes in gene expression. To what extent these changes manifest at the protein level has never been investigated on a proteome-wide scale. Here, we have studied HDACi-treated cells by large-scale mass spectrometry based proteomics. We show that HDACi treatment affects primarily the nuclear proteome and induces a selective decrease of bromodomain-containing proteins (BCPs), the main readers of acetylated histone marks. By combining time-resolved proteome and transcriptome profiling, we show that BCPs are affected at the protein level as early as 12 h after HDACi treatment and that their abundance is regulated by a combination of transcriptional and post-transcriptional mechanisms. Using gene silencing, we demonstrate that the decreased abundance of BCPs is sufficient to mediate important transcriptional changes induced by HDACi. Our data reveal a new aspect of the mechanism of action of HDACi that is mediated by an interplay between histone acetylation and the abundance of BCPs. Data are available via ProteomeXchange with identifier PXD001660 and NCBI Gene Expression Omnibus with identifier GSE64689.

The acetylation pattern of histone tails determines how tightly or loosely the DNA is wrapped around nucleosomes and thus controls the accessibility and the transcription of genes (1). An increase in acetylation leads to an instability of the nucleosomes and a higher accessibility of the DNA (2). Histone acetyltransferases and deacetylases (HDACs) are the two main classes of enzymes that regulate the acetylation of histones and other proteins. In humans, 18 different HDACs have been identified and grouped into four classes. Despite their name, HDACs target not only histones but also other proteins, including transcription factors, transcriptional coregulators, enzymes involved in DNA repair, and chaperones (3). Bromodomains recognize acetylated lysine residues and are the main readers of histone tail signatures (4). Bromodomain-containing proteins (BCPs)^1^ are multidomain proteins that recruit various factors and protein complexes to the acetylated sites. They thus mediate several biological processes, including chromatin remodeling (5), transcription regulation (6), E3 protein ligase activity (7), and histone methyl- and acetyl-transferase activities.

The expression of various HDACs is elevated in different types of cancer. Because of their central role in transcriptional control, they are considered excellent drug targets (8). Several HDAC inhibitors (HDACi) have been successfully tested in cancer therapy and over 20 HDACi compounds have entered various phases of clinical development. Vorinostat, a hydroxamate, was the first HDACi approved as drug for cutaneous T-cell lymphoma in 2006 (9). Vorinostat is used for patients not responding to prior systemic treatments or with recurrent cutaneous T-cell lymphoma and has a response rate of 30% (10). Meanwhile, another HDACi (romidepsin) has been approved for treatment of cutaneous T-cell lymphoma and peripheral T-cell lymphoma.

HDACi cause hyperacetylation of histone tails and positively contribute to anticancer therapy by inducing various pathways. Previous studies revealed that HDACi arrest growth and cell cycle by increasing the expression of the cyclin-dependent kinase inhibitor 1 (CDKN1A, encoding the protein p21) that interrupts the interaction of cyclins with cyclin-dependent kinases (11). Furthermore, HDACi stimulate both the intrinsic apoptotic pathway, by up-regulating pro-apoptotic and down-regulating anti-apoptotic factors (12) and the extrinsic apoptotic pathway through an increased expression of death receptors and ligands (13). Oxidative stress has also been proposed as a potential mechanism of action of HDACi via an increase of reactive oxygen species and subsequent damage of mitochondria (14). However, it has also been shown that HDACi can function as neuroprotective agents by reducing oxidative stress (15, 16).

Although HDACis directly modify the epigenetic landscape, large-scale genomic studies of different cancer cell lines have shown that only 7 to 10% of the expressed genes are differentially regulated upon treatment (17, 18). However, how these complex effects induced by HDACi treatment are reflected at the proteome level remains poorly understood.

Compared with hematological neoplasms, the efficacy of HDACi in solid tumors is much lower, particularly in monotherapeutic approaches, although overexpression of HDACs is also frequently observed in solid malignancies and associated with a poor clinical outcome (19). Furthermore, *in vitro* and *in vivo* studies could demonstrate that individual HDACs are key mediators of tumor cell survival and tumorigenicity in *e.g.* lung, breast, colon, and cervical carcinoma cells (19). A randomized phase III trial revealed that the combination of HDACi and chemotherapy in patients with advanced cervical cancer leads to a significant advantage in progression-free survival over the chemotherapy treatment alone (20). Encouraged by these studies, we chose the cervical cancer cell line HeLa to analyze the proteome-wide effects of two classes of chemically distinct HDACi: the hydroxamates Trichostatin A (TSA) and Vorinostat and sodium butyrate (NaB). By combining subcellular fractionation with mass-spectrometry-based shotgun proteomics, we show that HDACi induce major alterations of the nuclear proteome where more than 25% of the detected proteins was significantly affected. In contrast, cytosolic proteins were largely unaffected. By integrating time-resolved transcriptomic and proteomic analysis, we show that transcriptional changes precede alteration of the proteome and that HDACi affect a number of chromatin regulators and transcription factors. In particular, HDACi consistently caused a selective depletion of the nuclear pool of several BCPs. Finally, we demonstrate for selected target genes that BPCs depletion by gene silencing is sufficient to recapitulate the transcriptional effects caused by HDACi. Our data thus reveal novel insights into how cells respond to the aberrant acetylation levels induced by HDACi and provide a rich resource for future studies that potentially will help to design improved cancer therapies.

## EXPERIMENTAL PROCEDURES

### 

#### 

##### Cell Culture and Drug Treatment

HeLa cells were grown in Dulbecco's modified Eagle's medium (DMEM)—low glucose 1 g/l (Sigma-Aldrich, Munich, Germany) supplemented with 10% heat-inactivated fetal bovine serum (FBS), penicillin streptomycin (PS), and 2 mm l-glutamine. Cells were allowed to attach to the culture dish for 24 h, then the drug, 2 μm Vorinostat (LKT Laboratories, Inc., St. Paul, MN), 1.5 μm TSA (Sigma-Aldrich), 300 mm sodium butyrate (Sigma-Aldrich) or 300 nm camptothecin (CPT) (Sigma-Aldrich), was added and cells were treated for 12 or 48 h. As a control, cells were treated with the same volume of ethanol or DMSO, in the case of CPT, for 12 or 48 h. The cells were washed twice with PBS for collection, while still attached to the culture dish, to exclude floating cells for all further experiments.

##### Thiazolyl Blue Tetrazolium Bromide (MTT) Assay

The cell metabolic activity was tested using the MTT assay. The cells were treated with HDACi concentrations ranging from 0.2 μm to 40 μm for 48 h in a 24 well plate. Afterward, cells were incubated with fresh medium, containing 0.5 mg/ml MTT (Sigma-Aldrich) for 1 h. The formazan crystals were dissolved by replacing the medium with DMSO/EtOH (1:2). The absorbance of the dissolved formazan crystals was measured at 570 and as a reference at 630 nm with a Biotek Synergy plate reader. R package “drc” was used to fit a dose-response curve and IC50 value was calculated based on the two-parameter log-logistic function (min: 0%, max: 100%) (21).

##### Western Blot

After 48 h of HDACi treatment, the cells or nuclei (after subcellular fractionation) were trypsinized and lysed in 4x Laemmli sample buffer and heated for 5 min at 95 °C. Standard protocols were used for SDS-PAGE, semidry transfer and Western blotting. As primary antibodies anti-acetylated-lysine (1:1,000) (Cell Signaling Antibody, Leiden, The Netherlands), anti-p21 (1:250) (Santa Cruz, Heidelberg, Germany), anti-glyceraldehyde-3-phosphate dehydrogenase (GAPDH) (1:1,000) (Sigma-Aldrich) and anti-lamin B1/B2 (1:5,000) (ImmuQuest, Seamer, United Kingdom) were used. The secondary antibodies were anti-rabbit IgG or anti-mouse IgM (1:5,000) (both Jackson ImmunoResearch, Suffolk, United Kingdom).

##### Subcellular Fractionation

The isolation of nuclei was performed according to (22) with the exception of a lower concentration sucrose cushion (2 m instead of 2.3 m) that was used for Vorinostat- and CPT-treated cells. For the cytosolic fraction, cells were collected and swollen in hypotonic buffer (50 mm Tris-HCl, pH 7.5, and protease inhibitors) like for nuclei isolation. Afterward, cells were ruptured by passing the suspension 8–9 times through a 27G needle. Crude nuclei were pelleted at 1,000 × *g* for 13 min at 4 °C. The resulting supernatant was spun at 100,000 × *g* for 30 min at 4 °C to eliminate the remaining membrane-associated organelles. The final supernatant, containing mainly soluble cytoplasmic proteins, was used for proteomics analysis. The cytosolic and nuclear fraction were lysed by addition of Rapigest (Waters, Saint-Quentin, France) and urea to a final concentration of 0.2% (v/v) and 4 m, respectively, and sonicated for 3 × 30 s. Samples were stored at −80 °C before being processed for mass spectrometry.

##### Protein Identification by Mass Spectrometry

The samples for mass spectrometry were prepared according to (22). For shot-gun experiments, samples were analyzed using a nanoAcquity UPLC system (Waters GmbH) connected online to a LTQ-Orbitrap Velos Pro instrument (Thermo Fisher Scientific GmbH, Schwerte, Germany). Peptides were separated on a BEH300 C18 (75 μm × 250 mm, 1.7 μm) nanoAcquity UPLC column (Waters GmbH) using a stepwise 145 min gradient between 3 and 85% (v/v) Acetonitril in 0.1% (v/v) Formic Acid. Data acquisition was performed by collision-induced dissociation using a TOP-20 strategy with standard parameters. Charge states 1 and unknown were rejected.

For the quantitative label-free analysis, raw files from the Orbitrap were analyzed using MaxQuant (version 1.2.2.5) (23). MS/MS spectra were searched against the human Swiss-Prot entries of the Uniprot KB (database release 2012–04, 23,270 entries) using the Andromeda search engine (24).

The search criteria were set as follows: Full tryptic specificity was required (cleavage after lysine or arginine residues, unless followed by proline); two2 missed cleavages were allowed; carbamidomethylation (C) was set as fixed modification; oxidation (M) and acetylation (protein N-term) were applied as variable modifications, if applicable; mass tolerance of 20 ppm (precursor) and 0.5 Da (fragments). The retention times were matched between runs, using a time window of 2 min. The reversed sequences of the target database were used as a decoy database. Peptide and protein hits were filtered at a false discovery rate of 1% using a target-decoy strategy (25). Additionally, only proteins identified by at least two unique peptides were retained (Table S1). For quantitative label-free analysis, protein abundance scores were calculated using the peptides.txt output of MaxQuant, as described in (26). Briefly, protein intensity was calculated from the summed intensity of all the proteotypic (unique) peptides matching that protein, normalized by dividing it by the molecular weight of the protein, and log_2_-transformed to obtain the final abundance score. All comparative analyses were performed using R version 3.0.1 (27). Only proteins identified in at least two replicates were considered when comparing protein abundances between control and drug treatments. To reduce technical variation, data were quantile-normalized using the preprocessCore library (28). Protein differential expression was evaluated using the limma package (29). Differences in protein abundances were statistically determined using the Student's *t* test moderated by the empirical Bayes method. Significant regulated proteins were defined by a cut-off of log_2_ fold change ≤ -1 or ≥ 1 and *p* value ≤ .01 (corresponding to a False Discovery Rate (FDR) < 0.05 in nuclear fractions after 48h and to a FDR < 0.12 after 12 h of drug treatment and a FDR < 0.11 in cytosolic fractions).

The mass spectrometry proteomics data have been deposited to the ProteomeXchange Consortium (http://proteomecentral.proteomexchange.org) (30) via the PRIDE partner repository (31) with the dataset identifier PXD001660.

##### Gene Expression Analysis

Total RNA was extracted using the RNeasy Plus Mini Kit (Qiagen, Hilden, Germany) following the manufacturer's protocol. Biotinylated cDNA was synthesized from 500 ng of total RNA using the Ambion WT Expression Kit (#4491974) according to manufacturer's instructions. Following fragmentation and labeling (WT Terminal Labeling and Controls Kit, Affymetrix, # 901524, High Wycombe, United Kingdom), cDNA was hybridized for 16 h at 45 °C on GeneChip Human Gene 2.0 ST Array. GeneChips were washed and stained in the Affymetrix Fluidics Station 450 and scanned using GeneChip *Scanner* 3000 7G. All microarray samples were preprocessed with Affymetrix Power Tools (APT, http://media.affymetrix.com/support/developer/powertools/changelog/index.html) and a robust microarray analysis normalization procedure was applied to generate log2-transformed expression measures for “meta” probe sets (transcript level as given by Affymetrix) (32). The quality of the samples was evaluated by checking if they have unusually higher/lower values of control measures such as “average raw intensity signal” (“pm_mean”) or “mean absolute deviation of the residuals” (“all_probeset_mad_residual_mean”). Next, detected above background method was used to identify probe sets with signals higher than the background distribution. The ‘meta’ probe sets were retained using a simple criterion (33) of whether more than half of the associated probes were detected (detected above background, *p* value < .05) in more than half of the replicates in any of the experimental conditions. After this filtering step, 36,065 out of 53,617 meta probe sets representing at least 14,632 known genes were retrieved for further analysis. In the next step, replicate samples were shown to cluster together using an unsupervised hierarchical clustering revealing no apparent outliers in the data set (Fig. S3*A*). Differentially expressed genes were identified for the comparisons of TSA and NaB treatments with the corresponding controls in 12 and 48 h (FDR adjusted *p* value < .01 and absolute log_2_ fold change >1) using limma package (34) available from Bioconductor (28). The data discussed in this publication have been deposited in NCBI's Gene Expression Omnibus (35) and are accessible through GEO Series accession number GSE64689 (http://www.ncbi.nlm.nih.gov/geo/query/acc.cgi?acc=GSE64689).

##### Depletion of Bromodomain-Containing Proteins by siRNA

Cells were allowed to attach to the dish for 24 h then transfected with PBRM1-specific siRNA (#s30400), BRD1-specific siRNA (#s24389), ZMYND11-specific siRNA (#s21153), CREBBP-specific siRNA (#s3495), and negative control no. 1 siRNA (#4390843) purchased from ambion by life technologies. 25 pmol of siRNA (final concentration of 10 nm) was transfected using 7.5 μl of Lipofectamine RNAiMAX, according to the manufacturer's protocol. The cells were incubated with the different siRNAs for 72 h.

##### Quantitative PCR (qPCR)

Total RNA was extracted using the RNeasy Plus Mini Kit (Qiagen) following the manufacturer's protocol. For cDNA synthesis, the QuantiTect Reverse Transcription Kit (Qiagen) was used. Quantitative real-time PCR (qRT-PCR) was used to examine the relative expression of PBRM1 (5′-tgccccatgacatgtattctc-3′, 5′-aatgtcattttctggtatttcagttg-3′), BRD1 (5′-cgatggggtgaggaacat-3′, 5′-acgcccttctgcttacagag-3′), ZMYND11 (5′-ttgccaggagatgagattga-3′, 5′-cacacgaaaacacaggtcaca-3′), CREBBP (5′-acaagcgaaaccaacaaacc-3′, 5′-aaagaagtggcattctgttgc-3′), AP2C (5′-tgagatggcagctaggaagaa-3′, 5′-agcagttctgtatgttcgtctcc-3′), RAI3 (5′-tctcaagaggaaatcactcaaggt-3′, 5′-gtgggatggagaattcctttt-3′), KDM1B (5′-ggctttttgccgtgttctat-3′, 5′-ttgtcatccagggtcctca-3′), FOXA1 (5′-agggctggatggttgtattg-3′, 5′-accgggacggaggagtag-3′), PLK1 (5′-gcaccgaaaccgagttattc-3′, 5′-gccagtccaaaatccccta-3′), MSL1 (5′-caggccaaggaaaaggagat-3′, 5′-cgttcaatccgagcaagg-3′), GEMI4 (5′-agtacctgcctgccttagatga-3′, 5′-ctgggtctgacctaagcctct-3′), p21 (5′-gtacttggagtattggggtctga-3′, 5′-cagtccaggccagtatgttacag-3′) internally normalized to the housekeeping gene lamin-B1 (LMNB1 (5′-aagcagctggagtggttgtt-3′, 5′-ttggatgctcttggggttc-3′)). For qRT-PCR analysis 25 ng of cDNA was used in a 20-μl reaction consisting of 11 μl of SYBR® Green PCR Master Mix (Applied Biosystems, Darmstadt, Germany), 10 μm forward and reverse primer, and water. Thermocycling was carried out using the StepOne^TM^ (Applied Biosystems). Relative mRNA levels were calculated using the ΔΔ*Ct* method (36). Significant changes were assessed by applying a Welch two sample *t* test on the Δ*Ct* values for treatment and control samples (37).

## RESULTS

### 

#### 

##### Determination of Optimal Drug Treatment Conditions

**Fig. 1.**
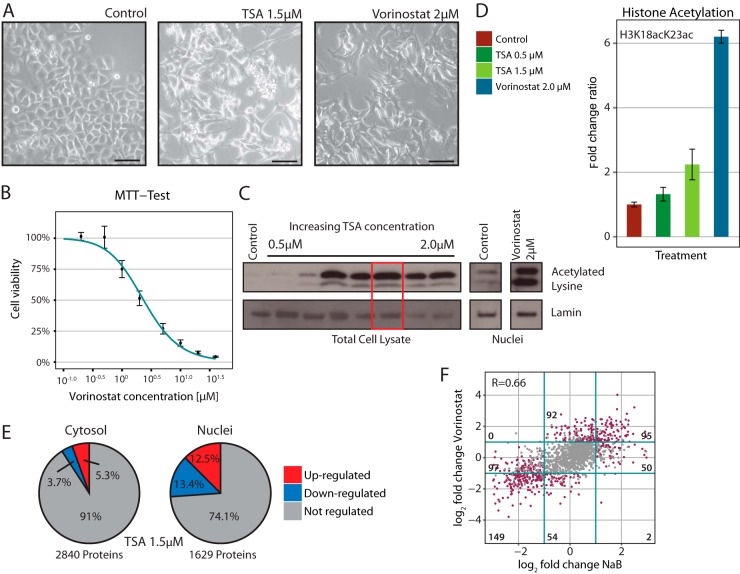
**Determination of optimal drug treatment conditions** (*A*) HeLa cells were cultivated for 24h and then treated with 1. 5 μm TSA or 2 μm Vorinostat for 48h. Control cells were treated with a respective amount of ethanol. Cells treated with HDACi show a typical morphology characterized by elongated protrusion. Scale bars are 100 μm. (*B*) MTT cell viability assay upon Vorinostat treatment. Vorinostat concentrations ranging from 0.2 μm to 40 μm were tested for three biological replicates. The data were expressed as percentage viability relative to untreated controls (set to 100%). (*C*) Western blotting of total cell lysate and nuclei with antibody against acetylated lysines (MW ∼18 kDa) shows an increase of acetylated histones following treatment with increasing TSA concentrations ranging between 0.5 μm and 2 μm. 50,000 cells or nuclei were loaded per lane, except the last two lanes of TSA treatment where only 28,000 cells were used. As a loading control an antibody against LaminB1/B2 (MW ∼66 kDa) was used. The red rectangle indicates a TSA concentration of 1.5 μm that was used for all the further experiments. (*D*) Increase of acetylation on histone 3 (H3K18acK23ac) measured in nuclei, using shotgun proteomics, upon HDACi treatment. All shotgun experiments were carried out in triplicate. (*E*) Pie chart showing the proportion of significantly regulated proteins with a *p* value ≤ .01 and a log_2_ fold change ≤ −1 or ≥ 1 in comparison to the total number of identified proteins upon TSA treatment. Major changes occur in the nuclear proteome while the majority of cytoplasmic proteins remain unaffected by the treatment. All shotgun experiments were carried out in triplicate. (*F*) Correlation of log_2_ fold changes induced by Vorinostat and NaB on the nuclear proteome. The correlation was calculated using the Spearman's rank correlation coefficient. Purple dots indicate proteins that have a *p* value ≤ .01 in at least one of the two conditions and a log_2_ fold change ≤ −1 or ≥ 1. The number of significant cases (“purple dots”) for each quadrant is indicated.

To investigate proteome remodeling induced by HDACi, we chose to treat HeLa cells with two different compounds of this drug class,TSA and Vorinostat. Both belong to the structural class of hydroxamates, and their main targets are HDACs of class I and II. We assessed the effectiveness of drug treatments by monitoring cell morphology, cell viability, and histone acetylation. At drug concentrations in the range of the previously determined IC_50_ dose of Vorinostat (38), the treatment induced characteristic morphological alterations of cells, such as the formation of elongated cellular protrusions (Fig. 1*A*) (39). To further prove the effectiveness of this dose, we probed the metabolic activity and viability of our cells using a MTT assay covering >2 orders of magnitude of Vorinostat concentrations (from 0.2 to 40 μm). The treatment with 2 μm Vorinostat showed reduction of cell viability close to 50% in comparison to untreated cells (Fig. 1*B*). To directly assay the activity of the drug, we monitored the increase in histone acetylation induced by HDACi by Western blotting and shotgun proteomics (Figs. 1*C* and 1*D*). We could detect an increase in histone acetylation following TSA treatment that saturated at a concentration of 1.5 μm after 48 h of treatment (Fig. 1*C*). For Vorinostat, saturation of histone acetylation was achieved at a concentration of 2 μm on the same time scale. Based on shotgun proteomics data (see below), we quantified the intensity of a specific double acetylated peptide on histone 3 (H3K18acK23ac). We detected up to a sixfold increase in the intensity of this acetylated peptide when cells were treated with Vorinostat (Fig. 1*D*). Similarly, treatment with TSA induced an increased level of H3K18acK23ac, although to a lesser extent than Vorinostat. We thus conclude that the chosen drug treatment conditions of 48 h with 1.5 μm TSA or 2 μm Vorinostat effectively inhibit histone deacetylase activity and induce the typical phenotypic changes that were previously reported.

##### HDACi Treatment Strongly Affects the Nuclear but not the Cytoplasmic Proteome

To monitor the impact of HDACi treatment on the nuclear and cytoplasmic proteome of treated cells we used subcellular fractionation and shotgun mass spectrometry using label-free quantification over three biological replicates (Fig. 1*E* and Fig. S1*A* and Table S2). The biological reproducibility of our experiments was assessed by calculating Spearman's rank correlation coefficient between protein intensities followed by hierarchical clustering, as shown in Fig. S1*B*. We cross-quantified 1,629 nuclear and 2,840 cytoplasmic proteins in TSA-treated cells as well as 1,821 and 2,880 proteins, respectively, in Vorinostat-treated cells. The cytoplasmic proteome remained largely unaffected, and only 9% of the identified proteins significantly changed their abundance. More pronounced changes occurred within the nuclear proteome with more than one-fourth (25.9%) of the quantified proteins being significantly affected. The changes induced by the two different HDACi were overall consistent, showing a positive correlation of *r* = 0.44 (Fig. S1*C*). In order to unequivocally link the observed effects to the mechanism of action of HDACi, we repeated the analysis of the nuclear proteome 48 h after treatment with sodium butyrate (NaB), a non-hydroxamic acid based HDACi, using the range of previously determined IC_50_ dose in HeLa cells (40). The effects of NaB were overall consistent and correlated to those induced by Vorinostat (Fig. 1*F*) and TSA (Fig. S1*D*) (*r* = 0.66 and *r* = 0.40, respectively), indicating that the majority of the proteomic changes derive from the shared mechanism of action of these compounds rather than from off-target effects. In total, 223 proteins were consistently identified as significantly regulated by these three compounds with 98 of them being significantly down- and 119 up-regulated. The other six proteins showed opposed fold changes. We thus conclude that HDACi induce a similar phenotype and common proteomic responses that primarily manifest in the nuclear proteome, but they also have more subtle compound-specific effects.

##### HDACi Treatment Causes a Massive Remodeling of the Cellular Abundance of Bromodomain-Containing Proteins

**Fig. 2.**
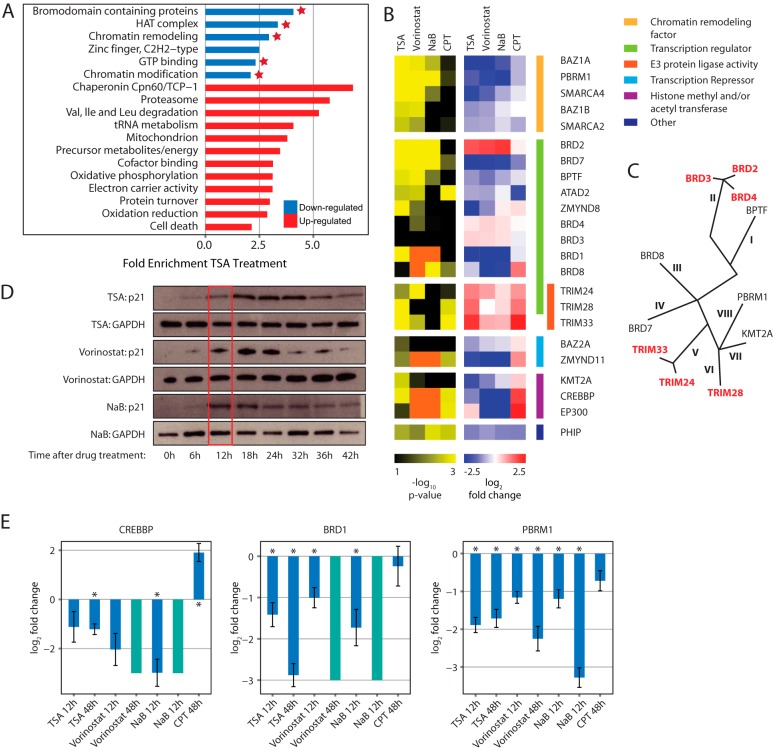
**HDACi treatment causes a massive remodeling of bromodomain containing proteins** (*A*) Gene ontology enrichment analysis of nuclear proteins affected by TSA treatment. Down-regulated proteins are significantly enriched in BCPs (fold enrichment ≥ 2, EASE Score (modified fisher exact *p* value) ≤ .1; only categories containing at least eight proteins are displayed). Enriched groups with a red star contain BCPs. (*B*) Heatmap showing the effect of HDACi and CPT treatment on BCPs. Three biological replicates per treatment were analyzed and displayed by their -log_10_ *p* value and log_2_ fold change. BCPs are grouped in functional groups. While the majority of the BCPs are significantly down-regulated, BCPs involved in protein degradation pathways are up-regulated by HDACi treatment. Orange squares in the *p* value heatmap indicate proteins that were identified exclusively and consistently in one sample group in all the three replicates but not in the other, and they were therefore considered as potentially regulated by the drug treatment. These cases were assigned an arbitrary log_2_ fold change of +3 (for proteins identified only after drug treatment) or -3 (for proteins identified only in control samples). (*C*) Phylogenetic tree of BCPs based on bromodomain sequence similarity (adapted from (51)). While down-regulation is apparent in almost all classes of BCPs, the up-regulated BCPs group into two clusters formed by BRD2, BRD3, and BRD4, and by the three E3 ubiquitin ligase-containing BPCs, respectively. (*D*) Western blot of total cell lysate with antibody against p21 (MW ∼18 kDa) shows an increased abundance of p21 following treatment with TSA (1.5 μm), Vorinostat (2 μm) and NaB (300 mm) already at 12 h (framed red). 50,000 cells were loaded per lane. As a loading control an antibody against glyceraldehyde-3-phosphate dehydrogenase (MW ∼36 kDa) was used. (*E*) Bar chart of the log_2_-fold changes of selected BCPs (CREBBP, BRD1, and PBRM1) as observed after HDACi and CPT treatment. BCPs depletion is induced by HDACi but not by CPT. All the reported values are averages of three biological replicates, and error bars indicate the standard error of the mean. Asterisks indicate significant cases. Cyan bars indicate proteins that were identified exclusively and consistently in control samples but were not detectable after drug treatment. These cases were considered as potentially regulated by HDACi, and they were assigned an arbitrary log_2_-fold change of −3.

To assess which pathways or cellular components are affected by HDACi, we first analyzed the enrichment of gene ontology terms in significantly regulated proteins for the nuclear fraction of treated cells (Fig. 2*A* for TSA and Fig. S2*A* for Vorinostat and Fig. S2*B* for NaB, respectively, Table S3). The gene ontology enrichment identified several classes of bromodomain-containing proteins (BCPs) that were reduced in abundance. In total, 23 out of 46 BCPs were cross-quantified, more than half of which showed a significant change of abundance upon drug treatment, the vast majority with reduced abundance (Fig. 2*B*). Most of the down-regulated BCPs are involved in chromatin remodeling, including components of the BAF and PBAF complexes such as SMARCA4 (BRG1) and PBRM1 (BAF180), transcription regulation, and repression (*e.g.* BRD7 and ZMYND11) as well as histone post-transcriptional modifications (*e.g.* CREBBP and EP300). In contrast, only six BCPs showed an opposing behavior, namely an up-regulation that was consistent across all three compounds, although of reduced extent in case of NaB (Fig. 2*B*). Interestingly, these six BCPs cluster into two distinct functional and phylogenetic classes (Fig. 2*C*) that share characteristic domain arrangements (Fig. S2*D*). Three of them, TRIM24, TRIM28, and TRIM33, have E3 protein ligase activity (41), while the other three BCPs (BRD2, BRD3, and BRD4) are known to function in transcriptional regulation (42). We thus conclude that HDACi treatment causes a massive remodeling of BCPs that predominantly results in reduced protein levels. Interestingly, the effect of HDACi appears to be linked to the functional and structural properties of the subclasses of BCPs.

In order to exclude that the changes in BCP levels result from secondary effects (*e.g.* drug toxicity), we repeated our proteomic analysis using an earlier time point upon treatment (12 h) that was selected on the basis of p21 protein induction, a known target of HDACi (Fig. 2*D*). Globally, the proteomic changes that manifested at 12 h were consistent with changes detected at 48 h although less pronounced in terms of fold change (Fig. S2*E*). For several BCPs, including CREBBP, BRD1, and PBRM1, we could detect already at 12 h a significant change in protein abundance that was, in general, increased after 48 h (Fig. 2*E*). This indicates that the regulation of BCPs is an early event following HDACi treatment and likely represents a primary effect of these compounds.

Finally, in order to test whether the regulation of BCPs is part of a more general response to the apoptotic stimuli induced by HDACi, we compared the proteomic profiles of HDACi to the one obtained using CPT, an inducer of apoptosis (43). Although we identified several alterations of the nuclear proteome that are common between HDACi and CPT, the changes induced by HDACi are globally distinct from the apoptotic signature (Fig. S2*F*). Only 63 of 278 (23%) proteins regulated by both classes of HDACi were also altered by CPT. BCPs in particular were largely unaffected by CPT treatment. Out of the 10 BCPs affected by TSA/Vorinostat and NaB (BAZ1A, PBRM1, BRD2, BRD7, BRD1, BRD8, ZMYND11, CREBBP, EP300, and PHIP), only the pleckstrin homology domain-interacting protein (PHIP) was consistently down regulated also by CPT (Fig. 2*B*). Other BCPs such as CREBBP, ZMYND11, and EP300 were significantly affected upon CPT treatment but regulated in opposite direction relatively to HDACi, namely up-regulated, further highlighting the specificity of the effect of HDACi on BCPs.

Taken together, these findings indicate that a general depletion of BCPs occurs specifically following HDACi treatment, presumably as a consequence of major remodeling of their target sites by histone tail hyperacetylation.

##### Reduction of Bromodomain-Containing Proteins Is Caused by Both Transcriptional and Nontranscriptional Regulation

**Fig. 3.**
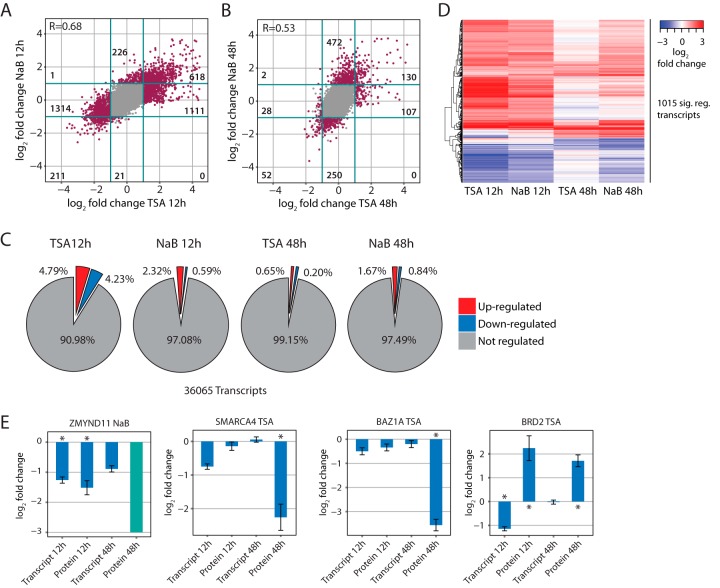
**Microarray gene expression analysis of cells treated with TSA and NaB for 12 h and 48 h.** TSA and NaB have consistent effects on the transcriptome as shown by the positive correlation of the log_2_ fold changes induced by two compounds after 12 (*A*) and 48 h (*B*). The correlation was calculated using the Spearman's rank correlation coefficient. Purple dots indicate significantly affected transcripts (adjusted *p* value ≤ .01 and log_2_-fold change ≤ −1 or ≥ 1, in at least one of the two conditions). The number of significant cases (“purple dots”) is shown for each quadrant. (*C*) HDACi affect only a small fraction of the transcripts. Pie charts showing the proportion of significantly regulated transcripts in comparison to the total number of quantified transcripts upon TSA or NaB treatment after 12 or 48 h. All microarray experiments were carried out in triplicate. (*D*) The effect of HDACi is more pronounced at 12 than 48 h at the transcriptome level. The heatmap shows the log_2_-fold changes of the 1,015 transcripts that were significantly affected by both TSA and NaB either at 12 or 48 h. 97% (986) of the transcripts show a consistent effect between TSA and NaB. The displayed values are averages of three biological replicates. (*E*) Bar chart showing the abundance of selected BCPs (ZMYND11, SMARCA4, BAZ1A, and BRD2) as log_2_-fold changes after HDACi treatment for 12 or 48 h. These BCPs show different combinations of transcriptional and post-transcriptional regulation. All the reported values are averages of three biological replicates and error bars indicate the standard error of the mean. Asterisks indicate significant cases. Cyan bar indicates a protein that was identified exclusively and consistently in control samples but were not detectable after drug treatment. This case was considered as potentially regulated by HDACi and it was assigned an arbitrary log_2_-fold change of −3.

To test if the reduced protein abundance of the majority of the BCPs arose from transcriptional or post-transcriptional regulation, we performed gene expression analysis at 12 and 48 h following treatment with TSA or NaB (Table S4). The effects of the two HDACi were globally consistent showing a positive correlation at both 12 and 48 h (*r* = 0.68 and *r* = 0.53, respectively) (Figs. 3*A* and 3*B*), and they affected a rather minor fraction of the transcriptome (<5%) (Fig. 3*C*). The biological reproducibility of our experiments was assessed by calculating Spearman's rank correlation coefficient between protein intensities followed by hierarchical clustering, as shown in Fig. S3*A*. In total, we identified 1,015 transcripts that are affected by both TSA and NaB (Fig. 3*D* and Table S4), 986 of which (97%) were consistently regulated having fold changes with the same sign. In contrast to the proteomic changes, the alterations of transcript level were generally more pronounced at 12 than 48 h (Fig. 3*D* and Figs. S3*B* and S3*C*), implying that they precede proteomic alterations.

For the many of the BCPs that we detected as regulated in the proteomic experiments, we identified corresponding changes in transcript abundance (Fig. S3*D* and Table S5). Consistent with the global effects on the transcriptome, changes in BCP transcript level were generally more pronounced at 12 h (Fig. S3*D*). However, the dynamics between transcript level and protein abundance varied among BCPs. In a certain subset of BCPs, both transcript and protein changes could be detected after 12 h (PBRM1, BRD1, ZMYND11, and CREBBP) while in others the change in transcript level preceded the alteration of protein abundance, which became apparent only after 48 h (*e.g.* for SMARCA4) (Fig. 3*E* and Figs. S3*D* and S3*E*). These variations might reflect different turnover rates. For two BCPs, namely BAZ1A and BRD2, protein abundance changes could not be explained by altered transcript level, suggesting the involvement of post-transcriptional mechanisms induced by HDACi. In the case of BRD2, we even detected significant down-regulation of its transcript at 12 h, while its protein abundance was increased at both 12 and 48 h after treatment with TSA and NaB (Fig. 3*E* and Fig. S3*E*). These data suggest that, although the reduced abundance of the majority of BCPs often derives from transcriptional regulation induced by HDACi, in some cases, post-transcriptional regulation also plays an important role.

##### Depletion of BCPs by Gene Silencing Recapitulates Transcriptional Changes Induced by HDACi

**Fig. 4.**
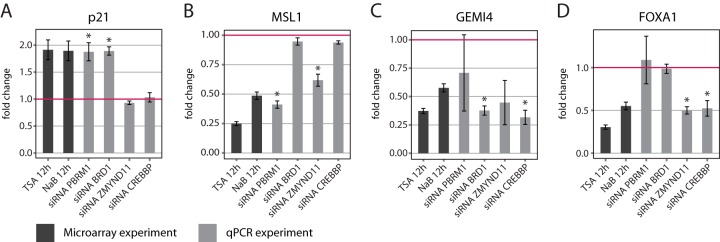
**Depletion of BCPs by RNA*i* is sufficient to mimic the effects of HDACi treatment onto selected transcripts** (*A*), (*B*), (*C*), and (*D*) Bar charts showing transcript abundances of selected target genes (p21, MSL1, GEMI4, and FOXA1) after siRNA-mediated depletion of four BCPs (PBRM1, BRD1, ZMYND11, CREBBP). Transcript levels were quantified by qPCR, and they are expressed as fold changes relatively to a scrambled siRNA control (set to one). For comparison, the fold changes induced by TSA and NaB after 12 h of treatment and quantified by microarray are displayed as dark gray bars. In all the cases shown, the individual depletion of two distinct BPCs induces a change in transcript level that is consistent and of similar effect size to the one induced by HDACi. All the reported values are averages of three biological replicates and error bars indicate the standard error of the mean. Asterisks indicate significant cases (Welch two sample *t* test *p* value < .05).

BCPs are known to affect transcription and chromatin structure, and their depletion occurs as early as 12 h after HDACi treatment (Fig. S3*D*). We thus wondered if BCP depletion alone might be able to induce at least some of the changes in the transcriptome that were induced by HDACi. We therefore individually depleted four BCPs by RNA*i* that were found consistently depleted by both HDACi classes after 12 h of treatment, namely PBRM1, BRD1, CREBBP, and ZMYND11 (Fig. S4*A*). We then selected eight putative target proteins that were significantly affected by HDACi at both the protein and transcript level (Table S5). These include: the polo-like kinase 1 (PLK1), the transcription factor FOXA1, the chromatin regulator male-specific lethal 1 homolog (MSL1), the retinoic-acid-induced protein 3 (RAI3), the transcription factor AP2C, the histone demethylase KDM1B, the spliceosome component gem-associated protein 4 (GEMI4), and p21. For each target protein, we measured transcript levels following depletion of BCPs using qPCR and compared the effect to HDACi treatment. Changes in transcript levels were measured relatively to a scrambled siRNA control. For half of the arbitrarily selected targets (four out of eight, namely p21, MSL1, GEMI4, and FOXA1), the depletion of one or more of the selected BCPs was sufficient to induce significant changes in transcript levels similar to those observed upon HDACi treatment (Figs. 4*A*–4*D*). In particular, the depletion of both PBRM1 and BRD1 induced a twofold increase of p21 transcript level (Fig. 4*A*), a well-established response to HDACi treatment (11). Interestingly, both PBRM1 and BRD1 repressed other target genes. PBRM1 drastically reduced the transcript levels of MSL1 (Fig. 4*B*) and PLK1 (although not to a significant level, *p* value .08) (Fig. S4*B*), while BRD1 repressed the expression of GEMI4 (Fig. 4*C*). Finally, the depletion of both CREBBP and ZMYND11 caused a decrease of the transcript level of FOXA1 (Fig. 4*D*). The remaining three targets (RAI3, KDM1B, and AP2C) were not affected by the depletion of the selected BCPs (Figs. S4*C*, S4*D*, and S4*E*). These data imply the potential existence of a specific regulatory link between the abundance of BCPs and the expression of target genes affected by HDACi.

## DISCUSSION

Changes in the epigenetic landscape are crucial for the onset and progression of cancer. Histone acetylation is one of the most critical epigenetic marks that regulate chromatin state and ultimately gene expression. A sophisticated molecular network composed of several writers (histone acetyltransferases), eraser (HDACs), and readers (BCPs) is responsible for fine-tuning the acetylation code on histone tails and other proteins. Aberrant levels of histone acetylation as well as altered expression of key regulators have been reported for several cancer, prompting the development of drug targeting this system, in particular HDACi. More recently, molecules targeting the bromodomain and extraterminal family of BCPs have been studied, and some are already tested in clinical trials, for the treatment of different types of carcinoma and inflammation (44). Here, we investigated the proteomics changes induced by two well-characterized HDACi classes and found that the most severe effects manifested in the nucleus, presumably as a consequence of the severe changes induced on chromatin structure (1, 45). The three tested compounds (TSA, Vorinostat, and NaB) affected the nuclear abundance of several BCPs in a consistent way, unraveling a novel regulatory circuit that links HDAC activity to the expression of the readers of acetylated lysines. Interestingly, different families of BCPs were differentially affected by HDACi treatment. HDACi generally induced a decrease in the abundance of BCPs across almost all types of domains. In contrast, the bromodomain and extraterminal domain containing BRD2 and, to a lesser extent, the related BRD3 and BRD4 were up-regulated. The majority of these changes was specific to HDACi treatment and not induced by the unrelated apoptosis inducer CPT. Interestingly though, a subclass of BPCs, including TRIM-28 and -33 were significantly up-regulated in response to TSA, Vorinostat, and CPT, but not NaB (Fig. 2*B*). This group of BCPs is likely to be involved in a more general response to apoptotic stimuli. Notably, TRIM-28 and -33 form a multiprotein complex together with TRIM-24 that has been shown to act as tumor suppressor, for example, in the context of hepatocellular carcinoma (46, 47).

**Fig. 5.**
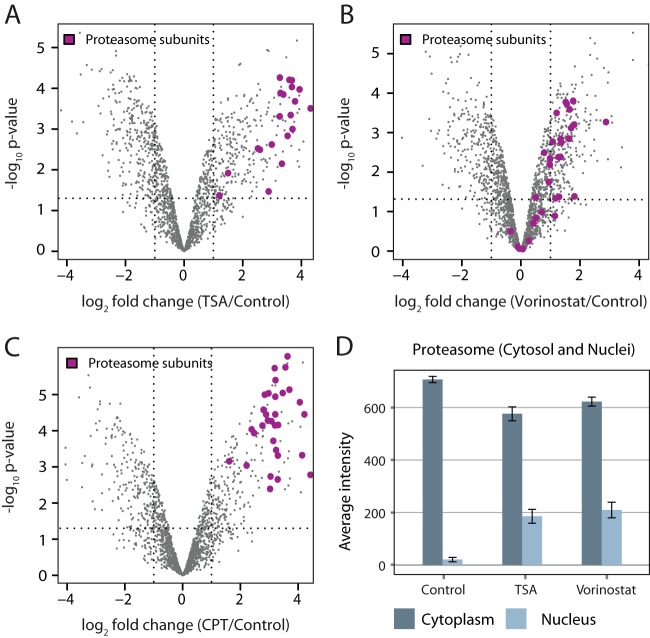
**The abundance of nuclear proteasome increases in response to both hydroxamate HDACis and the apoptosis-inducing drug CPT.** The abundance of proteasome subunits increases dramatically in the nuclear fraction after treatment with TSA, Vorinostat, and CPT. Volcano plots of all proteins quantified in HeLa nuclei in comparison to their respective control for TSA (*A*), Vorinostat (*B*), and CPT (C) treatment. Purple dots indicate the identified subunits of the proteasome. (*D*) Comparison of the proteasomal distribution within subcellular fractionated cells upon HDACi treatment using shotgun mass spectrometry. The measured intensity of the proteasome subunits was normalized to the number of cells or nuclei analyzed. Dark gray bars indicate the cytosolic, light gray bars the nuclear fraction. The nuclear-localized proteasome increased up to 10-fold in abundance following HDACi treatment, while in the cytosol the abundance of the proteasome was reduced by 19 and 12% by TSA and Vorinostat, respectively. This indicates that the increase in nuclear-localized proteasome is mediated by relocalization of the proteasome from the cytosol to the nucleus following drug treatment.

Our work reveals further alterations of the nuclear proteome that are common between HDACi and CPT and therefore likely represent general features of apoptosis or stress responses. In particular, we identified a dramatic increase of proteasome in the nuclear fraction (up to 10-fold) in response to TSA, Vorinostat, and CPT (Figs. 5*A*–5*C*, but not NaB; data not shown) that was mediated by a relocalization of part of the cytoplasmic pool to the nucleus (Fig. 5*D*). Relocalization of proteasome to the nucleus was previously observed in cell lines following glucose-starvation or hypoxia (48), and it might thus represent a universal stress response.

The time-resolved analysis of proteome and transcriptome allowed us to define the dynamics of transcript changes and their effect on protein abundance following exposure to HDACi. In agreement with previous reports (17, 18), we showed that the effects of HDACi on transcription are rapid events occurring within the first 12 h after treatment. In addition, we were able to show that many of these changes are transient and revert to nonsignificant levels after 48 h (Figs. S3*B* and S3*C*). Since the levels of histone acetylation induced by HDACi remain elevated over the 48 h of treatment (Fig. 1*C*), we speculate that many of the transcriptional changes induced by HDACi within the first 12 h are mediated by alterations of transcriptional regulators. It appears that the effect of HDACi occurs in two stages. In the first instance, a series of chromatin and transcriptional regulators, many of them containing bromodomains, are modulated in their expression, presumably in response to the aberrant level of histone acetylation induced. Then the change in abundance of these proteins has an inducing effect on a second group of transcripts. This hypothesis is supported by our ability to recapitulate the transcriptional changes induced by HDACi by depleting single BCPs. Particularly striking is the fact all four BCPs tested had an effect on at least one of the selected target genes that was consistent and of comparable effect size to the one induced by HDACi (Figs. 4*A*–4*D*). In order to check whether the effects of HDACi on the BPCs that were transcriptionally regulated, as well as candidate target genes, could also be detected in other cell lines, we compared the transcriptome changes observed in HeLa cells to data sets for other cancer cell lines that are available in public databases (49). In agreement with a previous report (50), the effects of HDAC inhibition that we described for HeLa cells are common across cancer cell lines (Fig. S5), and they might thus represent a universal response underlying the mode of action of these compounds. These data might imply that HDACi affect the transcription of these genes through BCP depletion, but further experiments would be required to firmly establish a mechanistic link.

In summary, our work provides a time-resolved proteomic and transcriptomic view of the effect of HDACs inhibition in mammalian cells, and it reveals the existence of a functional link between the level of lysine acetylation, the nuclear abundance of its readers, the bromodomain-containing proteins, and the transcriptional changes induced by HDACi. Some of these findings might therefore contribute an important base for the improvement of HDACi-associated therapeutic strategies in solid tumors.

## Supplementary Material

Supplemental Data

## Footnotes

^1^ The abbreviations used are:

BCP: bromodomain-containing protein
BRD: bromodomain
CPT: Camptothecin
DMSO: dimethyl sulfoxide
HDAC: histone deacetylase
HDACi: histone deacetylase inhibitor
MTT: thiazolyl blue tetrazolium bromide
NaB: sodium butyrate
NET: N-terminal extra terminal
PCR: polymerase chain reaction
qPCR: quantitative polymerase chain reaction
TSA: Trichostatin A.
