# Supplementary material for: Histone Deacetylase Inhibitors (HDACi) Cause the Selective Depletion of Bromodomain Containing Proteins (BCPs)
Source: Mol Cell Proteomics. 2015 Mar 9;14(5):1350–60. doi: 10.1074/mcp.M114.042499 (PMC4424404; doi:10.1074/mcp.M114.042499)
Supplement: Supplemental Data [file supp_14_5_1350__index.html]

Histone deacetylase inhibitors cause the selective depletion of bromodomain containing proteins — Histone Deacetylase Inhibitors (HDACi) Cause the Selective Depletion of Bromodomain Containing Proteins (BCPs) — How HDACi Affect the Proteome of a Cell — Supplemental Data 

# Histone Deacetylase Inhibitors (HDACi) Cause the Selective Depletion of Bromodomain Containing Proteins (BCPs)

## Supplemental Data

**Files in this Data Supplement:**

- Supporting Information - Supporting Information
- Supplemental Table 1 - Protein Identification
- Supplemental Table 2 - Protein Quantitation: Summary statistics of limma of mass spectrometry analysis - Nuclear fraction treated with TSA, Vorinostat, NaB or CPT for 12 or 48 h - Cytosolic fraction treated with TSA or Vorinostat for 48h
- Supplemental Table 3 - GO Enrichment analysis of TSA, Vorinostat, NaB and CPT
- Supplemental Table 4 - Transcript Quantitation: Summary statistics of limma of microarray analysis - Cells treated with TSA or NaB for 12 or 48h
- Supplemental Table 5 - Combination of protein and transcript quantitation
