# Supplementary material for: Histone Deacetylase Inhibitors (HDACi) Cause the Selective Depletion of Bromodomain Containing Proteins (BCPs)
Source: Mol Cell Proteomics. 2015 Mar 9;14(5):1350–60. doi: 10.1074/mcp.M114.042499 (PMC4424404; doi:10.1074/mcp.M114.042499)
Supplement: Supplemental Data [file supp_M114.042499_mcp.M114.042499-1.pdf]

Supporting Information (SI)

Supporting Figure Legends

Figure S1: Determination of optimal drug treatment conditions and reproducibility of proteomic measurements

(A) Pie chart showing the proportion of significantly regulated proteins with a p-value ≤ 0.01 and a $\log_2$ fold change ≤ -1 or ≥ 1 in comparison to the total number of identified proteins upon Vorinostat treatment. Major changes occur in the nuclear proteome while the majority of cytoplasmic proteins remain unaffected by the treatment. All shotgun experiments were carried out in triplicate. (B) The reproducibility of proteomic measurements was assessed by calculating Spearman's rank correlation coefficient for the nuclear fraction treated with TSA, Vorinostat, NaB, and their controls for 48 h. Biological replicates for the same sample group are highly correlated and form distinct clusters when analyzed by unsupervised hierarchical clustering. (C) and (D) show the correlation of $\log_2$ fold changes induced by TSA and Vorinostat and TSA and NaB, respectively. The correlation was calculated using the Spearman's rank correlation coefficient. Purple dots indicate proteins that have a p-value ≤ 0.01 and a $\log_2$ fold change ≤ -1 or ≥ 1 in at least one of the two conditions. The number of significant cases ("purple dots") is shown for each quadrant.

Figure S2: HDACi induce specific changes to the nuclear proteome

(A) GO (Gene Ontology) enrichment analysis (fold enrichment ≥ 2, EASE Score (modified fisher exact p-value) ≤ 0.1; only categories containing at least 8 proteins are displayed) of nuclear proteins affected by Vorinostat treatment for 48 h. Groups marked with a red star contain BCPs. (B) and (C) the same as (A) but for NaB and CPT, respectively. (D) Domain structure of significantly up regulated BCPs. TRIM24,

TRIM28 and TRIM33 have an E3 protein-ubiquitin ligase activity while BRD2, BRD3 and BRD4 share a similar domain organization characterized by two bromodomains and a NET domain, involved in protein binding. (E) The effect of HDACi is more pronounced at 48 as compared to 12 hours at the proteome level. The heatmap shows the $\log_2$ fold changes of the 278 proteins that were significantly affected by both TSA and NaB either at 12 or 48 hours. 93% (259) of the proteins show a consistent effect between TSA and NaB. For comparison, the fold changes induced by the apoptosis-inducing drug CPT are shown. The displayed values are averages of three biological replicates. (F) HDACi induce specific changes to the nuclear proteome. Venn diagram of significantly regulated proteins after treatment with TSA, NaB, Vorinostat either at 12 or 48 hours, and CPT after 48h. The majority of the proteins regulated by both HDACi classes (215 out of 278, 77%) is not affected by CPT treatment.

Figure S3: Microarray gene expression analysis of cells treated with TSA and NaB for 12 h and 48 h

(A) The reproducibility of microarray experiments was assessed by calculating Spearman's rank correlation coefficient for replicate samples obtained from cells treated with TSA or NaB for 12 or 48 h and their respective controls. Biological replicates for the same sample group are highly correlated and form distinct clusters when analyzed by unsupervised hierarchical clustering. (B) and (C) The effects of HDACi are more pronounced at 12 hours upon treatment and decrease at 48 hours. Plot showing the relationship between fold changes at 12 and 48 hours for transcripts significantly regulated 12 hours upon treatment (adjusted p-value ≤ 0.01 and $\log_2$ fold change ≤ -1 or $\geq$). For the majority of up- (B) and down- (C) regulated transcripts the effect of HDACi decreases after 48h. Purple dots represent significantly affected transcripts and black lines connect the fold changes for the same transcript at 12 and 48 hours. (D) Heatmap summarizing the effect of TSA and NaB treatment after 12 and 48 h on the transcript and protein levels of BCPs. Three biological replicates per treatment were analyzed and displayed by their $-\log_{10}$ p-value and $\log_2$ fold change. (E) Bar chart showing the abundance of selected BCPs (ZMYND11, SMARCA4, BAZ1A, and BRD2) as $\log_2$-fold changes after HDACi treatment for 12 or 48

h. These BCPs show different combinations of transcriptional and post-transcriptional regulation. All the reported values are averages of three biological replicates and error bars indicate the standard error of the mean. Asterisks indicate significant cases.

Figure S4: Efficient depletion of BCPs by RNA*i*

(A) Bar chart of knockdown efficiency after siRNA mediated depletion of selected BCPs (PBRM1, BRD1, ZMYND11, CREBBP). Transcript levels were quantified by qPCR and they are expressed as fold changes relatively to a scrambled siRNA control (set to 1). (B) – (E) Bar charts showing transcript abundances of selected target genes (PLK1, RAI3, KDM1B and AP2C) after siRNA mediated depletion of four BCPs (PBRM1, BRD1, ZMYND11, CREBBP). Transcript levels were quantified by qPCR and they are expressed as fold changes relatively to a scrambled siRNA control (set to 1). For comparison, the fold changes induced by TSA and NaB after 12 h of treatment and quantified by microarray are displayed as dark gray bars. In the shown cases, the depletion of BPCs could not recapitulate the changes in transcript level induced by HDACi to statistical significance, suggesting that the regulation of these genes must be mediated by other factors than the selected BPCs. All the reported values are averages of three biological replicates and error bars indicate the standard error of the mean.

Figure S5: Common effects of HDACi across cancer cell lines

The heatmap visualizes the expression changes of 4 regulated BCPs and their putative target genes from four distinct human cell lines (HeLa; MCF7, breast adenocarcinoma; PC3, human prostate; HL60, promyelocytic leukemia, microarray dataset from The Connectivity Map treated with multiple HDACi compounds). In agreement with previous reports, HDACi-induced expression changes were shown to be similar across cell types.

Supporting Figures

Figure S1

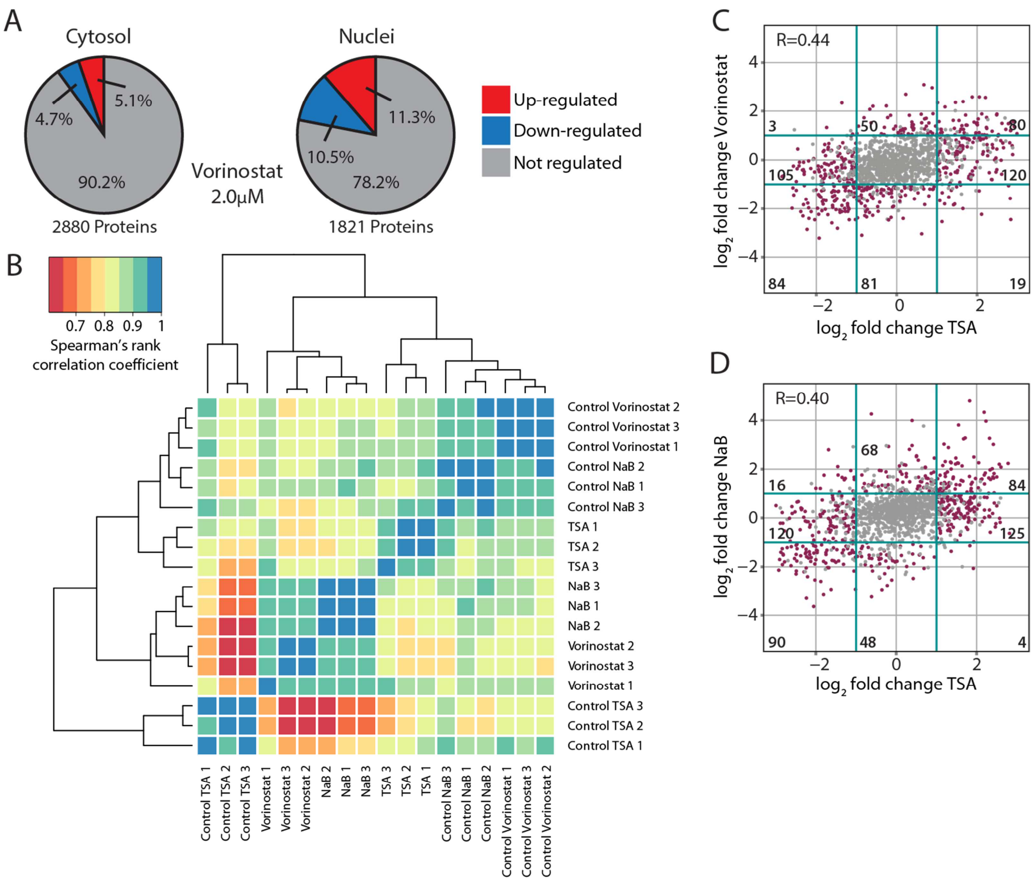

Figure S2

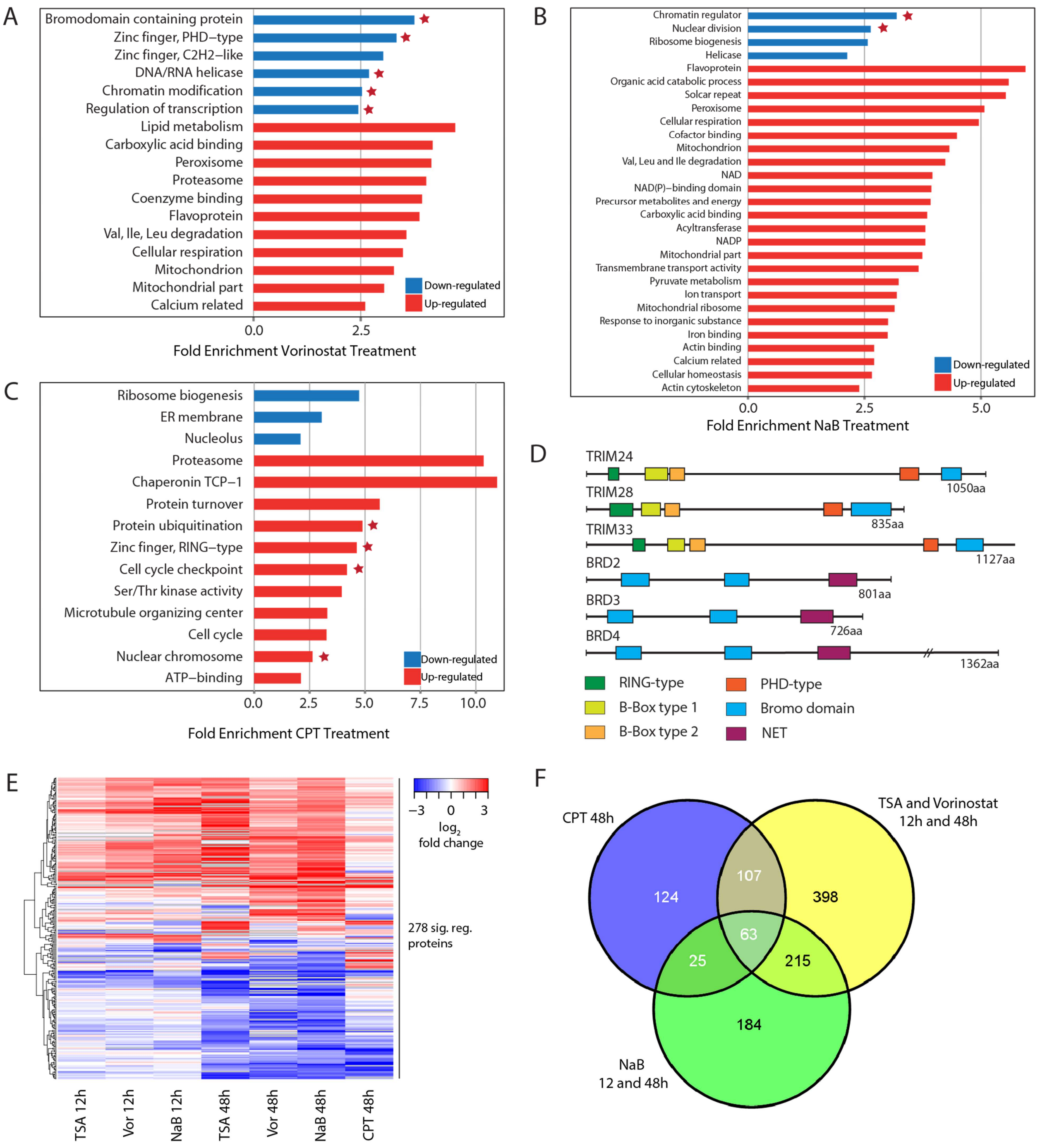
A
Bromodomain containing protein
Zinc finger, PHD–type
Zinc finger, C2H2–like
DNA/RNA helicase
Chromatin modification
Regulation of transcription
Lipid metabolism
Carboxylic acid binding
Peroxisome
Proteasome
Coenzyme binding
Flavoprotein
Val, Ile, Leu degradation
Cellular respiration
Mitochondrion
Mitochondrial part
Calcium related
Down-regulated
Up-regulated
0.0
2.5
Fold Enrichment Vorinostat Treatment
B
Chromatin regulator
Nuclear division
Ribosome biogenesis
Helicase
Flavoprotein
Organic acid catabolic process
Solcar repeat
Peroxisome
Cellular respiration
Cofactor binding
Mitochondrion
Val, Leu and Ile degradation
NAD
NAD(P)–binding domain
Precursor metabolites and energy
Carboxylic acid binding
Acyltransferase
NADP
Mitochondrial part
Transmembrane transport activity
Pyruvate metabolism
Ion transport
Mitochondrial ribosome
Response to inorganic substance
Iron binding
Actin binding
Calcium related
Cellular homeostasis
Actin cytoskeleton
Down-regulated
Up-regulated
0.0
2.5
5.0
Fold Enrichment NaB Treatment
C
Ribosome biogenesis
ER membrane
Nucleolus
Proteasome
Chaperonin TCP–1
Protein turnover
Protein ubiquitination
Zinc finger, RING–type
Cell cycle checkpoint
Ser/Thr kinase activity
Microtubule organizing center
Cell cycle
Nuclear chromosome
ATP–binding
Down-regulated
Up-regulated
0.0
2.5
5.0
7.5
10.0
Fold Enrichment CPT Treatment
D
TRIM24
1050aa
TRIM28
835aa
TRIM33
1127aa
BRD2
801aa
BRD3
726aa
BRD4
1362aa
RING-type
B-Box type 1
B-Box type 2
PHD-type
Bromo domain
NET
E
−3
0
3
log2 fold change
278 sig. reg. proteins
TSA 12h
Vor 12h
NaB 12h
TSA 48h
Vor 48h
NaB 48h
CPT 48h
F
CPT 48h
TSA and Vorinostat 12h and 48h
NaB 12 and 48h
124
107
398
63
25
215
184

Figure S3

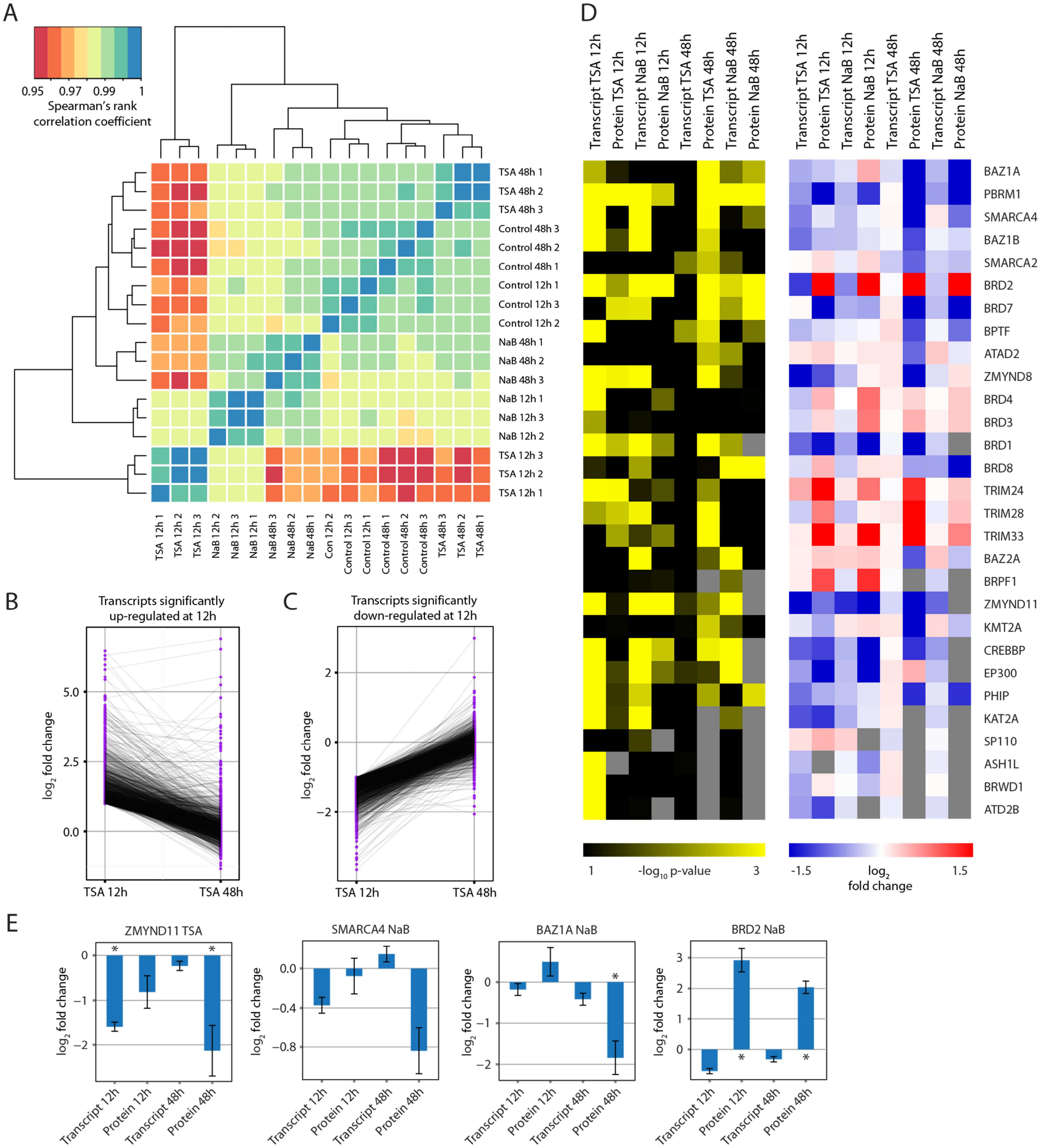
A
0.95
0.97
0.99
1
Spearman's rank correlation coefficient
B
Transcripts significantly up-regulated at 12h
C
Transcripts significantly down-regulated at 12h
log2 fold change
TSA 12h
TSA 48h
D
-log10 p-value
log2 fold change
BAZ1A
PBRM1
SMARCA4
BAZ1B
SMARCA2
BRD2
BRD7
BPTF
ATAD2
ZMYND8
BRD4
BRD3
BRD1
BRD8
TRIM24
TRIM28
TRIM33
BAZ2A
BRPF1
ZMYND11
KMT2A
CREBBP
EP300
PHIP
KAT2A
SP110
ASH1L
BRWD1
ATD2B
E
ZMYND11 TSA
SMARCA4 NaB
BAZ1A NaB
BRD2 NaB
Transcript 12h
Protein 12h
Transcript 48h
Protein 48h

Figure S4

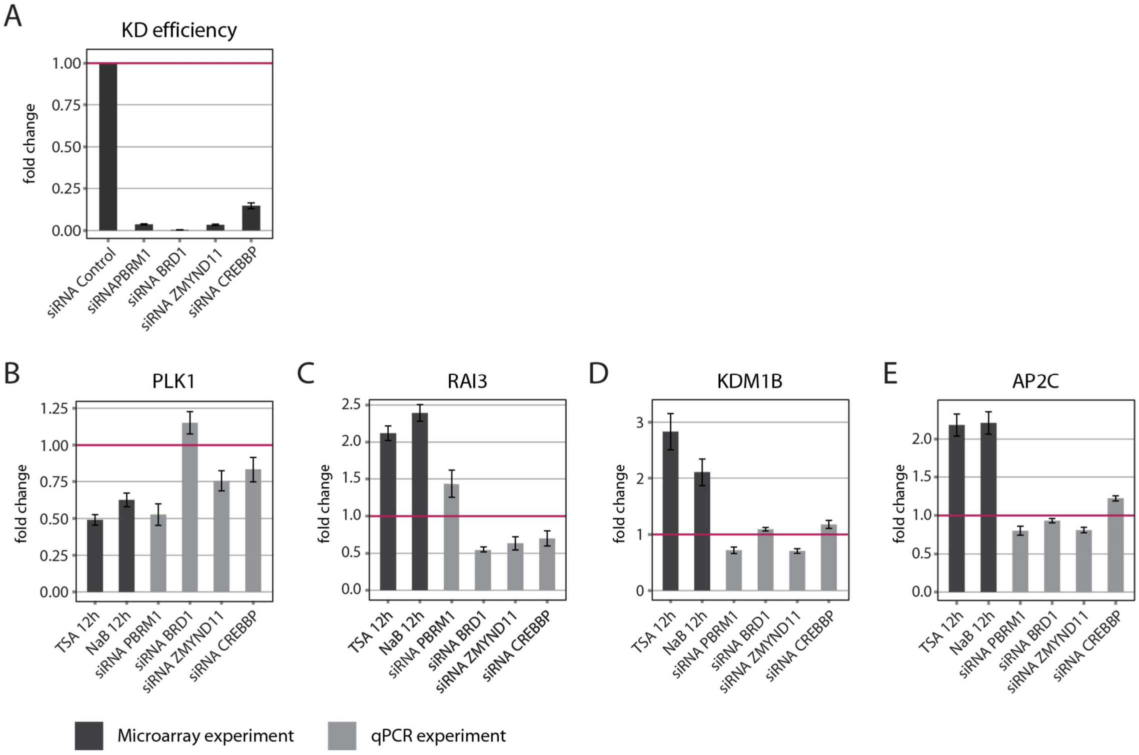
A
KD efficiency
fold change
siRNA Control
siRNAPBRM1
siRNA BRD1
siRNA ZMYND11
siRNA CREBBP
B
PLK1
C
RAI3
D
KDM1B
E
AP2C
TSA 12h
NaB 12h
siRNA PBRM1
siRNA BRD1
siRNA ZMYND11
siRNA CREBBP
Microarray experiment
qPCR experiment

Figure S5

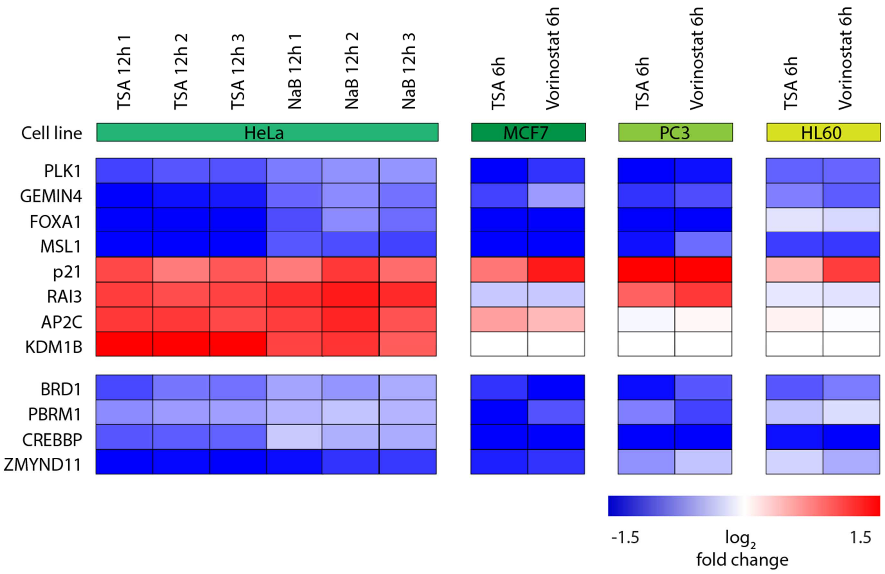
TSA 12h 1
TSA 12h 2
TSA 12h 3
NaB 12h 1
NaB 12h 2
NaB 12h 3
TSA 6h
Vorinostat 6h
TSA 6h
Vorinostat 6h
TSA 6h
Vorinostat 6h
Cell line
HeLa
MCF7
PC3
HL60
PLK1
GEMIN4
FOXA1
MSL1
p21
RAI3
AP2C
KDM1B
BRD1
PBRM1
CREBBP
ZMYND11
-1.5
log2 fold change
1.5

Supporting Datasets

- Table S1: Protein Identification
- Table S2: Protein Quantitation: Summary statistics of limma of mass spectrometry analysis
    - Nuclear fraction treated with TSA, Vorinostat, NaB or CPT for 12 or 48 h
    - Cytosolic fraction treated with TSA or Vorinostat for 48h
- Table S3: GO Enrichment analysis of TSA, Vorinostat, NaB and CPT
- Table S4: Transcript Quantitation: Summary statistics of limma of microarray analysis
    - Cells treated with TSA or NaB for 12 or 48h
- Table S5: Combination of protein and transcript quantitation
